# A framework for player movement analysis in team sports

**DOI:** 10.3389/fspor.2024.1375513

**Published:** 2024-08-05

**Authors:** Stan Parker, Grant Duthie, Sam Robertson

**Affiliations:** ^1^ Institute for Health and Sport (IHeS), Victoria University, Melbourne, VIC, Australia; ^2^ High Performance Department, Western Bulldogs Football Club, Melbourne, VIC, Australia; ^3^ School of Exercise Science, Australian Catholic University, Strathfield, NSW, Australia; ^4^ Sports Performance, Recovery, Injury and New Technologies (SPRINT) Research Centre, Australian Catholic University, Melbourne, VIC, Australia

**Keywords:** spatiotemporal analysis, physical movement, complex systems, analytics, open questions, working framework

## Abstract

Player movement is a fundamental component of evaluating performance in most team sports. Movement can be evaluated across multiple scales, referring to the function of anatomical structures through various planes of motion or an individual regulating their field position based on the movement of opposition players. Developments in commercially available tracking systems have afforded end users the ability to investigate the spatiotemporal features of movement in fine detail. These advancements, in conjunction with overlaid contextual information, have provided insights into the strategies adopted by players in relation to their movement. Understanding movement beyond its semantic value allows practitioners to make informed decisions surrounding performance evaluation and training design. This investigation proposes a framework to guide the analysis of player movement within team sports environments. The framework describes how operational standards for assessing movement can be designed in reference to theory and a set training philosophy. Such practice allows for the spatial and temporal complexities within team sports to be described and could potentially lead to better-applied outcomes through greater interdisciplinary collaboration and an improved holistic understanding of movement. To inform its development, this study evaluates the current research and identifies several open questions to guide future investigations.

## 1 Introduction

Understanding the features of player movement within team sports is a key component in athlete monitoring, performance evaluation, and training design processes (1). Determining what movement is and how to measure it effectively are fundamental requirements for developing strategies to improve sporting performance. Player movement is often defined and measured at varying levels of complexity across team sports and high-performance environments (2, 3). Developing a framework that acknowledges this complexity would allow for greater collaboration between disciplines by ensuring a shared language, potentially leading to better-applied outcomes.

Player movement can be measured across multiple scales, for example, the kinematic analysis of an anatomical structure's function through various planes of motion or positional descriptions of an individual in response to team tactical behaviour (4). In team-based invasion sports, movement is increasingly viewed through a spatiotemporal lens. Spatiotemporal data typically include the location of individuals or events with reference to space and time (5). Spatiotemporal features of movement can be described using one-dimensional derivatives of displacement, such as velocity, acceleration, and their angular properties; two-dimensional representations of manoeuvrability, such as tortuosity (5, 6); or three-dimensional depictions of limb kinematics (7), which are increasingly being used for *in situ* technique analysis, as well as various adjudication applications such as semi-automated offside assessment in football (8). As such, identifying a technology solution capable of measuring the intended movement feature with appropriate validity and reliability is paramount to successful evaluation.

Tracking systems such as Global Navigation Satellite Systems (GNSS), Local Positioning Systems (LPS), and Optical Tracking Systems (OTS) have provided the means to capture spatiotemporal information within indoor and outdoor team sports competitions. Such solutions provide positional data (longitude and latitude coordinates) and aggregated descriptors of movement, such as total running volume and maximal velocity. These systems often contain inertial measurement units (IMUs), which include a triaxial accelerometer, a triaxial gyroscope, and a triaxial magnetometer. IMUs have been used to assess kinematic movements such as stride length, flight time, and ground contact time, as well as acceleration metrics and the automatic detection of match phases (9, 10). A “player load” or “dynamic load” metric is an example of triaxial resultant information derived from an IMU being operationalised by a technology provider (11). Developments to these operating systems have seen capturing rates evolve from 1 to 100–300 Hz (9, 12, 13). In addition, computer vision research has brought about optical tracking algorithms that adopt deep learning models to produce ball or player-tracking solutions (14). Adoption of optical tracking solutions in sports varies due to unique sport-specific requirements. For example, greater revenue and salary cap confinements in the National Basketball Association (NBA), combined with controlled playing conditions (indoor environment, fixed camera operating systems, and fewer players to track), have granted relative access to the sport (15). Contrastingly, in Australian football, varying field sizes across the competition require significant camera infrastructure and therefore limit the viability of such a system (14).

This review aims to provide a framework for analysing players’ physical movement within team sports. To inform its development, this study evaluates the current research and identifies several open questions to guide future investigations.

## 2 Contextualisation of movement

To understand player movement beyond its semantic value, research has assessed the influence of various contexts. The constraint-led approach (CLA) provides an appropriate convention to categorise contexts previously used to measure sporting performance (2, 16). Constraints refer to the boundaries by which movement strategies emerge (2) and are categorised as individual, task, or environmental (17). Individual constraints refer to contextual factors that describe physical and technical attributes, historical descriptions of experience, and states of cognition (2). Task constraints describe rules and instructions and are related to the task goal (2, 18). Environmental constraints are external to the organism and refer to the physical or sociocultural conditions surrounding performance (2, 18). Table 1 provides a selection of constraints used to provide context to player movement in team sports. For example, understanding individual movement behaviour during offensive and defensive periods of play allows performance staff to make informed decisions regarding training load prescription, while understanding the influence of numerical imbalances may assist coaches in making more informed decisions when their team is influenced by injuries or player exclusions.

**Table 1:** Summary of various contexts reported in the literature for the analysis of player movement in team sports.

Group	Type	Constraint	Example
Match events	Task	Movement value	Soccer (1, 19), Australian football (20)
	Task	Decision-making value	Basketball (21), Australian football (22, 23), soccer (24), futsal (25)
	Task	Field location	Australian football (26–28)
	Task	Possession phase	Australian football (29–31), soccer (32), rugby league (33), rugby union (34), basketball (35)
	Task	Collective behaviour	Australian football (29, 36), soccer (37–39), basketball (40)
	Task	Technical involvements	Australian football (28, 41, 42), soccer (43, 44)
	Task	Ball-in-play time	Australian football (45)
	Task	Playing time	Australian football (46–49), soccer (50, 51), basketball (52)
	Task	Playing position	Rugby league (53), soccer (54), basketball (35), American football (55), ice hockey (56)
	Task	Spatial occupancy	Australian football (57–59), soccer (60–62)
Individual	Individual	Playing experience	Australian football (63–66), soccer (67–69), netball (70)
	Individual	Peak demands	Rugby league and Australian football (71–73), rugby league (71, 73), American football (74), field hockey (75), basketball (76, 77), netball (78), lacrosse (79)
	Individual	Aerobic capacity	Australian football (80, 81), rugby league (82, 83), ice hockey (56)
	Individual	Strength and power	Australian football (84), rugby league (85), ice hockey (86), soccer (87)
	Individual	Gender	Soccer (88, 89)
	Individual	Muscle fibres	Australian football (90)
	Individual	Training load	Australian football (80, 91), rugby league (92, 93)
	Individual	Starting vs substitute	Soccer (94, 95), rugby league (96),
	Individual	Sleep	Rugby league (97)
	Individual	Subjective wellness	Australian football (98), field hockey (99), American football (100)
	Individual	Fatigue	Australian football (101–104), soccer (105)
	Individual	Warm-up duration	Futsal (106)
Match Context	Task	Numerical advantage	Australian football (107), soccer (108)
	Task	Team/Opposition quality	Soccer (109), rugby league (110, 111)
	Task	Game significance	Australian football (112), rugby league (113), American football (114), netball (115)
	Task	Score differential (match outcome)	Australian football (29, 116), soccer (117, 118), ice hockey (119)
	Task	Team synergy	Basketball (120), soccer (121), Australian football (122), futsal (123)
	Task	Team formation	Soccer (124)
	Task	Team strategy	Soccer (125, 126), field hockey (127)
	Task	Number of rotations	Futsal (128)
	Environment	Weather	Australian football (129), soccer (130)
	Environment	Match location	Australian football (131), soccer (132)
	Environment	Fixture congestion	Rugby league (133), soccer (134)
	Environment	Travel required	Rugby league (135)
	Environment	Playing surface	Soccer (136, 137)

## 3 Current opportunities

Research will likely continue to build on the body of work mentioned above, largely driven by rapid technology enhancements. While this alone will probably lead to incremental improvements in the understanding of player movement in team sports, access to more data also has the potential to lead to misuse and/or misinterpretation. As a result, additional attention being paid to the areas below will likely have an accelerative and perhaps more fundamental impact comparatively.

Technological improvements to commercially available tracking systems have afforded the end user the ability to answer related questions in greater detail. For example, near real-time access to limb tracking enables precise rule adjudication and technique analysis. Prior to these recent improvements, the utilisation of tracking data to date has often been limited to the use of discrete representations of movement. For example, speed is captured at 10 Hz via GNSS but is often reported as an average speed over the duration of a drill. This is likely partly due to the lack of computational power to deliver outputs in a timely manner. These representations include describing the movement as a volume of running within predetermined arbitrary velocity thresholds, counts of efforts above set thresholds, and measures of intensity derived by volume and duration metrics (107, 138, 139). Such representations of movement do not consider the emergent patterns within team sports and reduce human behaviour to isolated numerical values (140, 141). While value models and rule adjudication techniques in sports routinely use continuous movement data, their benefits are yet to be fully realised in routine practice. Discrete representations of movement are easily understood and acted upon, whereas continuous data require greater computation capacity but allow for the complexity within the sport to be accounted for. For example, previous examinations of maximal periods of movement have provided a guide to infer training intensity, whereas the automation of skeletal tracking data from competition would support the development of three-dimensional representations of movement during different components of the game (e.g., fast break, one-on-one situations, etc.). This actualises the potential for real-time coaching, omnipresent monitoring, more precise training prescription, and enhanced broadcast fan engagement features.

Another potential accelerator in this area relates to the implementation of theoretical frameworks to underpin player movement analysis. The adoption of an overarching, organisation-wide theoretical framework has been suggested as a method of unifying action across disciplines (16). Such a framework would offer an approach to combining operational methodologies and techniques alongside theory and a set training philosophy (2, 142, 143). It could also serve to coordinate and unify activity, redirect resource allocations, stimulate coherent interdisciplinary communication, and promote a more holistic understanding of sports performance (5, 144). The collaboration of subdiscipline specialists allows for analysis to be communicated through consistent language, which could influence the transfer of knowledge and ultimately improve performance (41). Within sports science, the integration of a framework allows scientific methodologies to reflect the broader training philosophy of organisations. Within the strength and conditioning discipline, it underpins exercise selection and progression. Within the coaching discipline, it underpins training design and performance evaluation, while in the medical discipline, it underpins rehabilitation design and physical screening.

Historically rooted in the physics literature, complex systems theory has recently been adopted by sports practitioners to underpin the analysis of movement (62, 121, 145, 146). This theory offers a framework through which a system’s stability, variability, and transition between fluid states can be described (147). Its adoption would be advantageous to sports performance, as this process-oriented philosophy embraces the complex constructs that operate within the team-sporting performance (147). Although all sports performance staff may benefit from its adoption, analysts and coaches are examples of two beneficiaries of complex systems research. The analysis of collective movement behaviour has enabled analysts to describe opposition ball movement methods and train accordingly, while the documentation of constraints on performance has permitted coaches to manipulate constraints in training to promote learning outcomes (148, 149). Complex systems theory describes athletes and teams as a complex adaptive system (CAS) whose behavioural properties continuously evolve in response to changing environments (12) and are unable to be understood outside of its context (150). In team-based sports, the theory recognises that individuals and teams regulate tactical behaviour in response to changing match constraints. It considers that control of tactical behaviour is distributed among different players and that individuals have the capacity to learn (4, 41, 151). Previous investigations have utilised a CAS approach to conceptualise team synergy in various sporting environments (13, 62, 121). Recent efforts to enhance interpretability have manifested in updated definitions and visual depictions of the 16 defining features of CAS theory (152). Complex systems theory provides a framework in which data analysis techniques can reference theory in the analysis of movement features. Table 2 provides an outline of the CAS features, their definitions, an analytical method that aligns with each feature, examples from the literature, and their proposed influence on practice.

**Table 2:** Matrix describing the 16 defining features of a complex systems theoretical approach and the analytical method through which they can influence practice.

Complex systems feature	Definition	Analytical methodology	Example from the literature	How does it influence practice?
Feedback	When the output of a process influences an input to accelerate or suppress change	Decision tree classification	Shot classification (157)	An increase in running load during competition leads to soreness and reduced running loads in the following sessions
Emergence	New, unexpected properties that arise from the interactions of system components	Shannon entropy	Particles (158)	The development of novel ball movement patterns originating from a sustained coaching puts emphasis on defensive structures
Self-organisation	The autonomous organisation of subgroups or individuals within a system	Centrality, flocking motion models	Ornithology (159)	An attacking unit alters their movement without coach input in response to going behind early in a match
Levers and hubs	Components of a system that have a disproportionate positive or negative influence on the system	Fuzzy cognitive mapping	Ecosystems (160)	A footballer is substituted and the match changes as a result, given their role as playmaker
Non-linearity	Relationships between inputs and outcomes are not linear, and a system response to change is not proportional to magnitude	Neural networks, hidden Markov models, decision tree classifier	Econometrics (161), pattern recognition (162)	There may be a sweet spot that exists in terms of physical effort—more running is not better *per se,* and its association with performance outcomes does not exist linearly
Domains of stability	A system gravitates towards a stable state until it is significantly perturbated. If perturbation is large enough, the state may change rapidly until a new stable state is found	Approximate entropy	Soccer (163)	Motor control patterns such as the walking gait or neck posture become very stable and harder to alter due to the frequency of use
Adaptation	Individuals or groups within a system are capable of learning, problem-solving, and anticipating future situations	Markov chain, estimation of distribution algorithms	Genetics/evolution (164)	The ability for an athlete to tolerate increased training load throughout the preseason period without becoming sore or requiring significant rest
Path dependency	Current and future actions depend on what happened beforehand	Time series analysis	Society development (165)	The inability to perform the full Olympic snatch movement without first mastering the overhead squad and having a suitable grip technique
Tipping points	The threshold point beyond which the behaviour of a system suddenly changes. Changes may take place slowly initially but suddenly increase in pace	Change point analysis	Australian football (101, 102)	An overload injury that occurs in response to high training volume
Change over time	Complex systems develop and change their behaviour over time. They are continuously evolving and adapting to inputs on the system	Time series analysis (motif discovery, bifurcation models, Markov chains)	Text string analysis (166)	The development of youth athletes, whereby substantial alterations to anthropometry are occurring throughout the duration of a competitive season
Open systems	The system is open and has external interactions. Examples could come in the form of information, personnel, and finances into or out of the system	Autoencoder neural network	Machine learning (167)	Disruptions to resourcing (coaches, facilities, financial remuneration) influence an athlete's support system and potentially their learning outcomes
Unpredictability	A complex system is inherently unpredictable. It is impossible to forecast the outcome from an input with precision	Randomness tests (Kolmogorov–Smirnov, chi-squared), variance	Soccer (1)	The opposition team applies a tactical strategy that is foreign and new to the team
Unknowns	Due to the dynamic and open structure of the system, it is inevitable to see unexpected effects of our interventions	Imputation techniques, interpolation/extrapolation, probabilistic approaches, maximum entropy	Engineering (168)	There are aspects to the subdisciplines within sports that are unknown yet influence outcomes. For example, the specific role genetics plays in certain behaviours or characteristics has not been measured and thus is unknown
Distributed control	Control of a system is distributed among many actors. No one actor has total control. Each actor may only have access to local information	Industrial master–slave protocol	Information technology/communication (169)	Although the coach imparts directives to the players, they are unable to complete the tasks for players during games and must therefore allocate responsibilities among the group
Nested systems	Within the complex system, there are subsystems that operate dependently or independently of the parent system	Hierarchical analysis, network analysis	Performance motivation (170)	Player within a positional group that operates within the team, working towards the same outcome with a focus on unique key performance indicators
Multiple scales and levels	Actors in complex systems can operate across different levels. For this reason, systems must be studied and understood from multiple perspectives simultaneously	Fuzzy control	Sport (171)	A technical coach, recruiter, and strength coach have various definitions of “talent”

More detailed data require not only greater computational but also analytical capacity. This permits moving beyond viewing sporting contexts in isolation and provides the ability to establish causal relationships between constraint groups or movement features (153). Such methodology offers a one-dimensional representation of movement while emphasising outcome behaviour and neglecting motives and causes (153). Recent investigations have adopted multivariate or time series approaches that address the temporal features and relatedness within constraints (18, 154–156). This allows the analytical process to be aligned to a feature within a CAS.

## 4 Proposed framework

The proposed framework below (Table 3) can assist practitioners and researchers in extracting the most from player movement data while developing operational standards that align with a set training philosophy. An example using a complex systems theory to evaluate the change in running intensity throughout a match has been included as a case study.

**Table 3:** Framework for the analysis of player movement in team sports.

Step 1: What question are you trying to answer?	At which point does a player's running intensity meaningfully change throughout a game?
Step 2: Adopt theoretical principles that reflect the organisations view of player movement.	Complex systems theory
Step 3: Clearly define the principles of relevance to answering the question.	Tipping point
Step 4: Which data sources do you have available to answer your question?	Player tracking data (Global Positioning Systems)
Step 5: Which metrics should be extracted from the data?	10 Hz representations of velocity, acceleration, and player load
Step 6: Can you apply any relevant context to provide further meaning?	Critical fatigue, chronic workload, environmental conditions, etc.
Step 7: What analytical tool can you use to investigate the defined question?	Change point analysis
Step 8: How does the chosen analytical tool align with the chosen theoretical principle?	Refers to the theoretical principle of tipping point and change over time. Change point analysis attempts to find a point along continuous data in which the values before and after are different
Step 9: Process and sustainability evaluation. For example, did the analysis lead to a productive decision and suitable action? How sustainable is the process and metric use?	The analysis identified the point at which intensity drops, allowing suitable action for player rotations. Player tracking data will always be available within a match environment

## 5 Open-ended research questions

Assuming progress in each of the above-mentioned areas, both practice and research are well-positioned to find solutions to further pervasive and impactful questions relating to player movement in team sports. A summary of some of these questions, many of which have already been posed in the literature, aimed at stimulating future investigations is provided as follows:

• OPEN QUESTION 1: Existing models for evaluating the difficulty of skilled actions have been made assessing the two-dimensional distance between ball carrier and receiver/target. Can a model be developed that accounts for three-dimensional space, the effect of ball spin and the role movement has on skill execution? (12) Such information would provide practitioners with a method of evaluating the complexities of skilled behaviour that could influence the training design and analysis processes.
• OPEN QUESTION 2: Investigations into player density have adopted commitment models to evaluate options afforded to players (20). How does physical capacity intersect with whether players should commit to a contest or not? Understanding the role physical capacity has on commitment would assist the development of density models that are reflective of human capacity. This would assist practitioners in making informed decisions around performance analysis and training design.
• OPEN QUESTION 3: Understanding an individual's intention is a key component to skill acquisition (172, 173). Individual intent is a constraint that shapes skill execution, and its identification allows coaches tactically to guide intention through instruction or drill design (16). Can spatiotemporal data provide a means to identify and evaluate a player's intentions and motivations? Whilst spatiotemporal data cannot identify underlying decision-making processes, it has the ability to identify relative risk tolerances, player passing option preferences and the quality of their decisions relative to other options. Understanding intent allows the quality of decision-making to be evaluated independently of skill execution and to guide the education process.
• OPEN QUESTION 4: High-performers are often referred to as having strong anticipatory skills (174, 175). How does the temporal component of decision-making influence outcomes and can spatiotemporal data provide insight into the temporal elements of decision-making risk and reward? Such information would infer the assessment of decision-making qualities and guide education and training processes.
• OPEN QUESTION 5: Spatiotemporal data actualises the potential for real-time omnipresent monitoring during training and match play. Can spatiotemporal data be used to identify meaningful fatigue before it occurs within open match play? Such information would allow coaches to optimise within-match rotation policies and potentially lead to better match outcomes.
• OPEN QUESTION 6: The definition of, and subsequent methods of measuring momentum in team sports is a largely unrefined research area. Providing a clear definition and a method of measuring such phenomenon allows end users to attempt to control its influence on match outcome. Can motion models infer momentum control and the techniques used to generate or retain it? Such information would help infer strategy and training design.
• OPEN QUESTION 7: Artificial intelligence (AI) models have the ability to consider information independently of human input and offer an alternative insight to a coach (176, 177). Understanding the differences in human–machine judgements within the coaching discipline is a fundamental component to building a symbiotic relationship between AI and sport moving forward. Can a model built on spatiotemporal data identify individual instantaneous critical fatigue, collective momentum, player form and opposition strategy, to inform which changes to player formation and strategy are required to improve the probability of match outcomes in real time? What are the associated risks involved? And how do these judgements differ from a coach's subjective model? Such models would operate as a decision-support system to enhance match outcomes, whilst concurrently informing the human–machine relationship within the coaching discipline.
• OPEN QUESTION 8: Within team-sport open-play scenarios, movement can be considered respective of the dynamic regulation of positioning in response to generating offensive or preserving defensive stability (107, 178). As such, training may foster environments that encourage desirable individual and collective spatial behaviour. How can spatiotemporal data infer individual or collective learning outcomes within open-play scenarios? Such information would assist with the evaluation of training outcomes and assessment of player development.
• OPEN QUESTION 9: As sporting organisations continually refine resourcing related to data architecture and processes, it is important to prioritise data sources that influence decision-making and performance. If we consider that physiological data could be used to compliment player tracking data, how would we establish the weight of each variable on performance? Generating a system to evaluate the influence of new data would allow organisations to prioritise resources, increase player performance, and enhance further innovation within the performance domain.

## 6 Conclusion

As long as technology continues to develop, the collection and analysis of spatiotemporal data will remain a common practice in high-performance sporting environments. Given this persistent nature, it is imperative that the analysis and description of player movement reflect not only the underlying physical quality but also the broader department philosophy rather than being reactive to resource availability. The merger of a set training philosophy with appropriate data analytical tools allows for the assessment of the complex features of movement in team sports. The stated open questions illustrate nascent areas of sports practice in which benefits could be actualised through the unification of theory and analytics. Such practice describes a methodology where data analysts operate within the confinements of a broader department-shared philosophy when analysing human physical movement. Ultimately, this promotion of interdisciplinary collaboration has the potential to achieve better-applied outcomes for players and organisations.
